# Pre-existing adaptive immunity to the RNA-editing enzyme Cas13d in humans

**DOI:** 10.1038/s41591-022-01848-6

**Published:** 2022-06-06

**Authors:** Xin-Zi Emily Tang, Shu Xuan Tan, Shawn Hoon, Gene W. Yeo

**Affiliations:** 1 Department of Physiology, Yong Loo Lin School of Medicine, National University of Singapore, Singapore, Singapore; 2 Molecular Engineering Laboratory, Institute of Molecular and Cell Biology, Agency for Science, Technology and Research, Singapore, Singapore; 3 Department of Cellular and Molecular Medicine, University of California, San Diego, La Jolla, CA USA; 4 Stem Cell Program, University of California, San Diego, La Jolla, CA USA; 5 Institute for Genomic Medicine, University of California, San Diego, La Jolla, CA USA

**Keywords:** Targeted gene repair, Applied immunology, Adaptive immunity

## Abstract

RNA-guided RNA-targeting nucleases, such as CRISPR–Cas13 proteins, have therapeutic potential for gene editing. Among Cas13d enzymes, Cas13d from the bacteria *Ruminococcus flavefaciens* (RfxCas13d) is of particular interest owing to its small size and high specificity. However, the existence of pre-existing immunity against RfxCas13d is unclear. In this study, we evaluated antibody and T cell responses to RfxCas13d in healthy donors using ELISA and T cell culture assays. We found RfxCas13d-reactive antibodies and CD4 and CD8 T cell responses in most donors, comparable to responses against Cas9 proteins from *Staphylococcus aureus* (SaCas9) and *Streptococcus pyogenes* (SpCas9). RfxCas13d-responding T cells could produce the inflammatory cytokines IFN-γ, TNF-α and IL-17. These findings should be taken into consideration in the development of RfxCas13d for therapy.

## Abstract (web summary)

Healthy individuals have antibodies and T cells that are reactive to the Cas13d protein from the bovine bacteria *Ruminococcus flavefaciens*, which may have implications for clinical testing of CRISPR–Cas13 gene editing approaches.

## Main

The discovery of CRISPR–Cas DNA-editing technologies has kickstarted the development of a plethora of therapeutics, adopting strategies such as eliminating disease-causing gene expression, repairing mutated genes, or adding new genes with new functions^1^. Many of these therapeutics use the most well-studied Cas9 enzymes from *S. pyogenes* (SpCas9) and *S. aureus* (SaCas9). However, several high-profile studies have reported widespread pre-existing antibody and T cell immune responses directed against these Cas enzymes in the general population^2–4^, likely due to previous exposure to the source bacteria. As such, these therapeutic strategies typically aim for transient expression of Cas enzymes to reduce the likelihood of immune-mediated toxicity that may compromise their therapeutic effects.

The use of RNA-editing technologies is also rapidly growing and may provide complementary capabilities. RNA-editing enzymes do not carry the risk of permanent, off-target changes in the genome and are ideal for situations in which homology-directed repair of DNA is not feasible, such as in terminally differentiated cells such as neurons and muscle cells. RNA editing could also provide tunable systems for modulating gene expression without complete elimination. Additionally, targeted messenger-RNA edits could be used to restore functions of large genes that cannot be easily replaced by gene therapy, or to modify alternative splicing, such as in spinal muscular atrophy^5^.

Cas13 enzymes are type VI CRISPR enzymes that are RNA-guided RNA-targeting nucleases. Several groups have demonstrated the use of Cas13 enzymes, either alone or in conjunction with other enzymes, to improve and restore functions in models of frontotemporal dementia^6^, spinal muscular atrophy^5^ and X-linked nephrogenic diabetes insipidus^7^. Unlike Cas9 enzymes, Cas13 enzymes are not limited by a protospacer flanking sequence, allowing many sites to be targetable. Their small gene and protein size enables easier packaging using adeno-associated viruses (AAVs) or lipid-based vectors, and they are thus highly suitable for therapeutic delivery. Among known Cas13 enzymes, RfxCas13d has been found to have the most robust knockdown efficiency while retaining high specificity and minimal off-target effects in mammalian cells^6,8^. RfxCas13d has also been successfully delivered in vivo to modulate cholesterol metabolism and improve Parkinson’s disease in mouse models^9,10^. Because RfxCas13d could be a promising therapeutic candidate, we asked whether the general population has pre-existing immunity directed against RfxCas13d, similar to SpCas9 or SaCas9.

Here, we were able to detect antibody responses to RfxCas13d, at a level comparable with responses to SaCas9 and SpCas9. We also detected CD4 and CD8 T cell proliferative responses and characterized the cytokine profile of T cells responding to RfxCas13d. The inflammatory responses directed against RfxCas13d are an important consideration in therapeutic use of RfxCas13d.

**Fig. 1:**
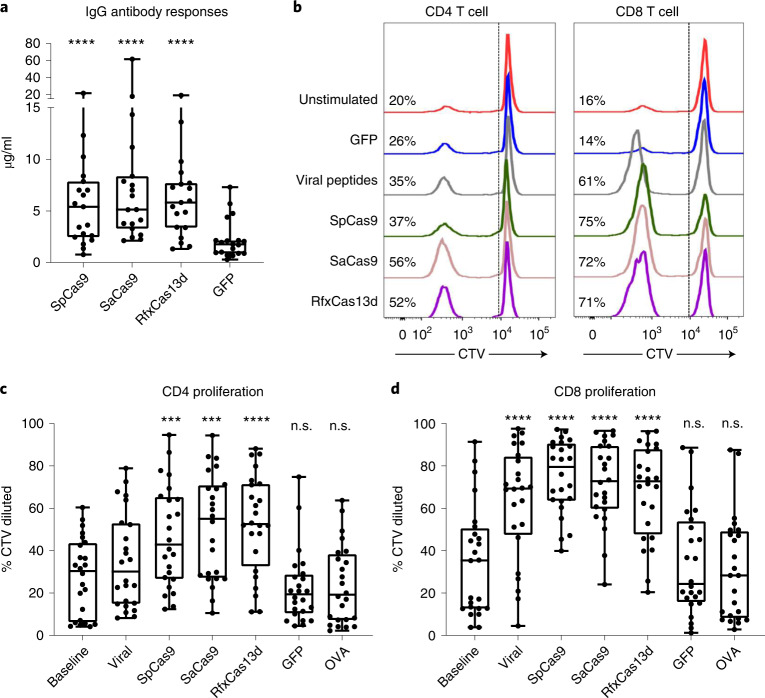
Antibody and T cell responses to RfxCas13d. **a**, IgG antibody responses to Cas proteins and GFP detected in human plasma in a direct ELISA assay (*n* = 19 biologically independent samples). **b**, Representative flow cytometric analyses of CD4 and CD8 T cell proliferation following stimulation with Cas proteins. Percentages indicate the frequency of proliferated cells within the CD4 or CD8 T cell subset. **c**,**d**, Aggregate data showing the frequency of proliferated CD4 (**c**) or CD8 (**d**) T cells (*n* = 24 biologically independent samples). Baseline indicates PBMCs cultured without antigen stimulation. Viral indicates stimulation with a pool of viral peptides. OVA, ovalbumin. Each data point represents one sample. Each sample was tested across all conditions shown in each graph. *P < 0.05; **P < 0.01; ***P < 0.001; ****P < 0.0001; n.s., not significant. One-way ANOVA with Dunnett’s multiple comparisons test on log transformed data using GFP as control (A). One-way analysis of variance (ANOVA) with Dunnett’s multiple comparisons test using baseline as control (**c**,**d**). For box plots, the center line indicates the median, box limits indicate the 25th and 75th percentiles, whiskers show minimum to maximum values and all individual data points are shown. Source data

**Extended Data Fig. 1:**
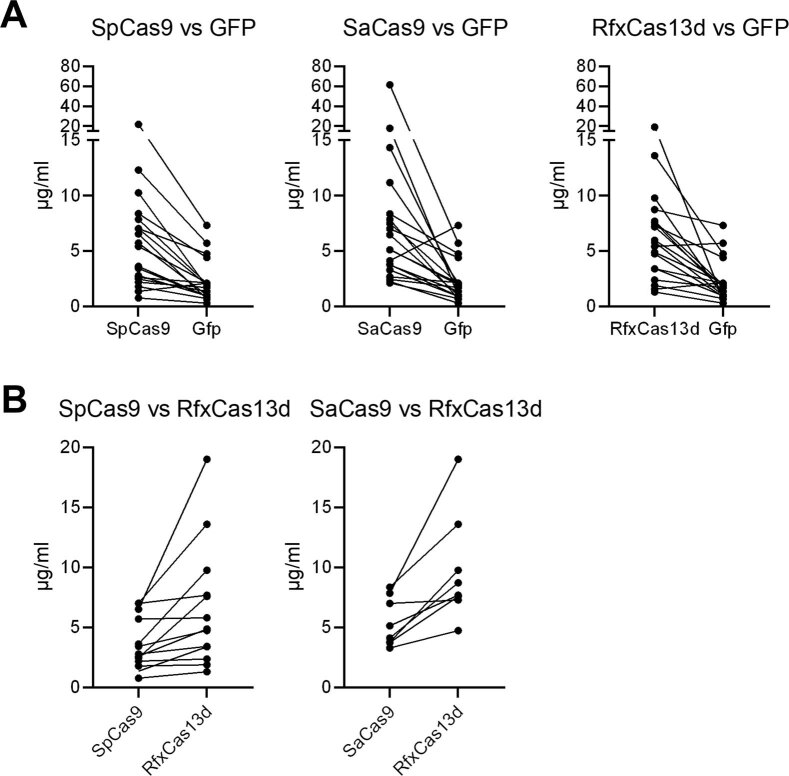
Paired comparisons of IgG antibody response between recombinant proteins. (**a**) Paired data comparing IgG antibody responses detected against each of the Cas proteins with GFP control protein. All analyzed samples shown (*n* = 19 biologically independent samples). (**b**) Paired data showing samples with higher IgG antibody responses against RfxCas13d compared to SpCas9 (*n* = 13) or SaCas9 (*n* = 8). Source data

To test whether human populations may have pre-existing humoral responses to RfxCas13d, we developed an ELISA assay to detect immunoglobulin G (IgG) antibodies in human plasma. For comparison, we expressed and purified recombinant SpCas9 and SaCas9 by the same procedures as those we used for RfxCas13d. We also used the same method to express and purify recombinant green fluorescent protein (GFP) as a negative control to detect other co-purified substances that may bind human IgG non-specifically. Of the 22 samples tested, 3 had higher background IgG binding to GFP than to the Cas proteins, and these were excluded from the analysis. For the remaining samples, the distribution of concentrations of IgG that bound RfxCas13d was comparable to the concentrations of IgG that bound SpCas9 and SaCas9, and was significantly higher than the concentrations of IgG that bound GFP (Fig. 1a). For RfxCas13d, 17/19 (89%) samples had IgG readings above the GFP background. For both SpCas9 and SaCas9, 18/19 (95%) samples had IgG readings above the GFP background (Extended Data Fig. 1a). In comparing antibody responses between Cas proteins, 13/19 samples had higher IgG readings for RfxCas13d than for SpCas9, and 8/19 samples had higher IgG readings for RfxCas13d than for SaCas9 (Extended Data Fig. 1b).

Our ability to detect antibody responses to RfxCas13d suggests that RfxCas13d-specific T cells are present because CD4 T cell help is necessary for production of high-affinity antibodies and IgG antibody class switching. To test for pre-existing T cell immunity against RfxCas13d, peripheral blood mononuclear cells (PBMCs) were cultured in the presence of recombinant RfxCas13d, and antigen-induced proliferation was measured. As a comparison, PBMCs were also stimulated with SpCas9 and SaCas9. Recombinant GFP similarly purified and certified endotoxin-free ovalbumin were used as negative controls. A pool of viral peptides from cytomegalovirus, Epstein–Barr virus, influenza and tetanus was used as a positive control for the assay. GFP- and ovalbumin-induced proliferation was comparable with that in unstimulated samples, demonstrating that our protein preparation did not contain other immunostimulatory substances. Antigen-induced proliferation was significantly higher than in unstimulated cultures for all 3 Cas proteins tested (Fig. 1b–d). We were able to detect antigen-induced proliferative responses in the CD8 T cell compartment in 23/24 (96%) samples for RfxCas13d, SpCas9 or SaCas9. For the CD4 T cell compartment, we detected antigen-induced proliferative responses in 22/24 (92%), 21/24 (88%) and 24/24 (100%) samples after exposure to SpCas9, SaCas9 or RfxCas13d, respectively. These data indicate that most individuals have existing immune recognition of RfxCas13d, comparable with that of SpCas9 and SaCas9.

**Fig. 2:**
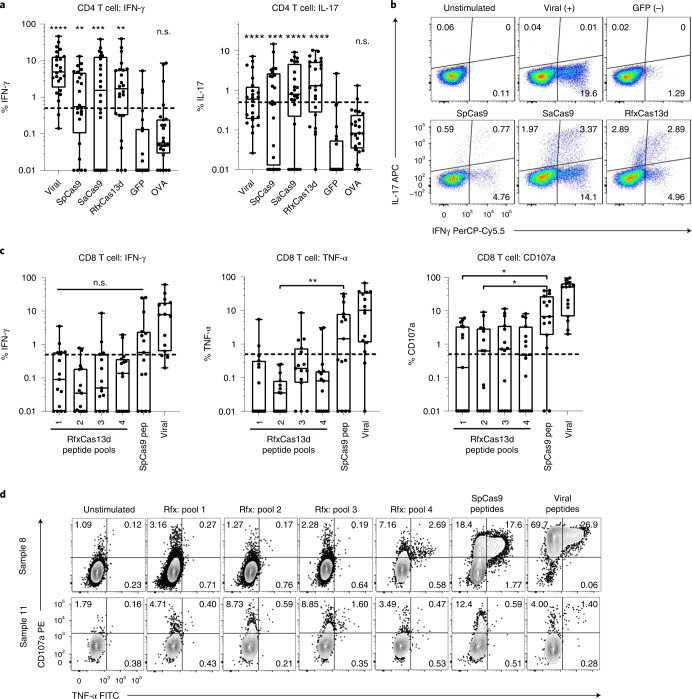
Cytokine profile of RfxCas13d-specific T cells. **a**,**b**, CD4 T cell responses following Cas protein restimulation on the last day of culture. **c**,**d**, CD8 T cell responses following overlapping peptide restimulation on the last day of culture. **a**,**c**, Aggregate responses are shown, with each data point representing one sample. (**a**, *n* = 23 biologically independent samples. **c**, *n* = 15 biologically independent samples.). Each sample was tested across all conditions shown. To ensure cytokine responses are indeed from cells expanded by antigen stimulation, each data point plots values after subtracting background responses from controls expanded with no antigen but re-stimulated with the tested antigen. Viral indicates stimulation with a pool of viral peptides. Differences in treatment conditions were tested using Friedman’s test with two-sided Dunn’s multiple comparisons test using GFP as the control (**a**) or across the SpCas9 and 4 RfxCas13d peptide pools (**c**). *P < 0.05; **P < 0.01; ***P < 0.001; ****P < 0.0001. For box plots, the center line indicates the median, box limits indicate the 25th and 75th percentiles and whiskers show minimum to maximum values. The dashed line indicates the limit of detection for antigen-specific responses. **b**, Representative flow cytometric analysis of CD4 T cell cytokine responses from one sample is shown. **d**, Flow cytometric analysis of CD8 T cell cytokine responses from two samples. Sample 8 shows TNF-α responses above background to RfxCas13d peptide pool 4, SpCas9 peptides and viral peptides. Sample 11 shows TNF-α responses above background to RfxCas13d peptide pool 3 and viral peptides. Source data

**Extended Data Fig. 2:**
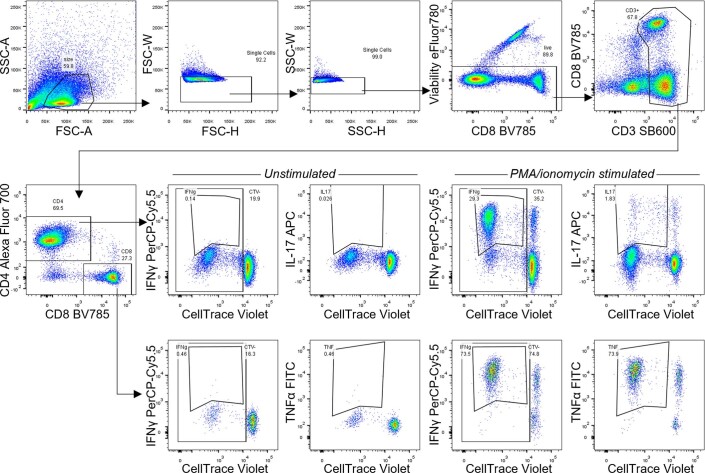
Gating strategy for flow cytometry.

**Extended Data Fig. 3:**
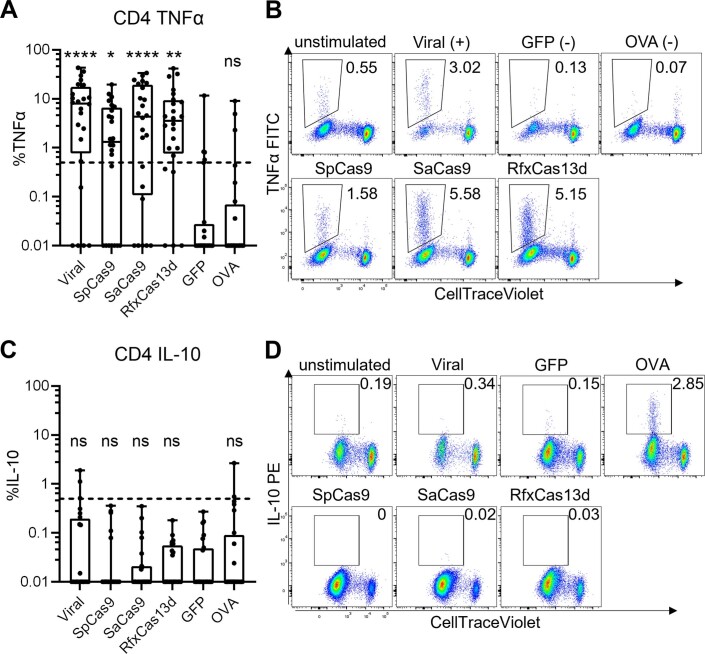
TNFα and IL-10 in CD4 T cells stimulated with recombinant Cas proteins. TNFα (**a**, **b**) and IL-10 (**c**, **d**) responses in CD4 T cells expanded with recombinant Cas proteins over 10 days and restimulated on the last day of culture. (**a**, **c**) Aggregate responses are shown, with each data point representing one sample. (*n* = 23 biologically independent samples). Each sample was tested across all conditions shown. To ensure cytokine responses are indeed from cells expanded by antigen stimulation, each data point plots values after subtracting background responses from controls expanded with no antigen but re-stimulated with the tested antigen. Differences in treatment conditions were tested using Friedman’s test with two-sided Dunn’s multiple comparisons test using GFP as the control. (ns, not significant, * p < 0.05, ** p < 0.01, *** p < 0.001, **** p < 0.0001). For box-plots, center line indicates median, box limits indicate 25^th^ and 75^th^ percentiles, whiskers show minimum to maximum value. Dashed line indicates the limit of detection for antigen specific responses. (**b**, **d**) Representative flow cytometric analysis of cytokine responses from one sample is shown. Source data

**Extended Data Fig. 4:**
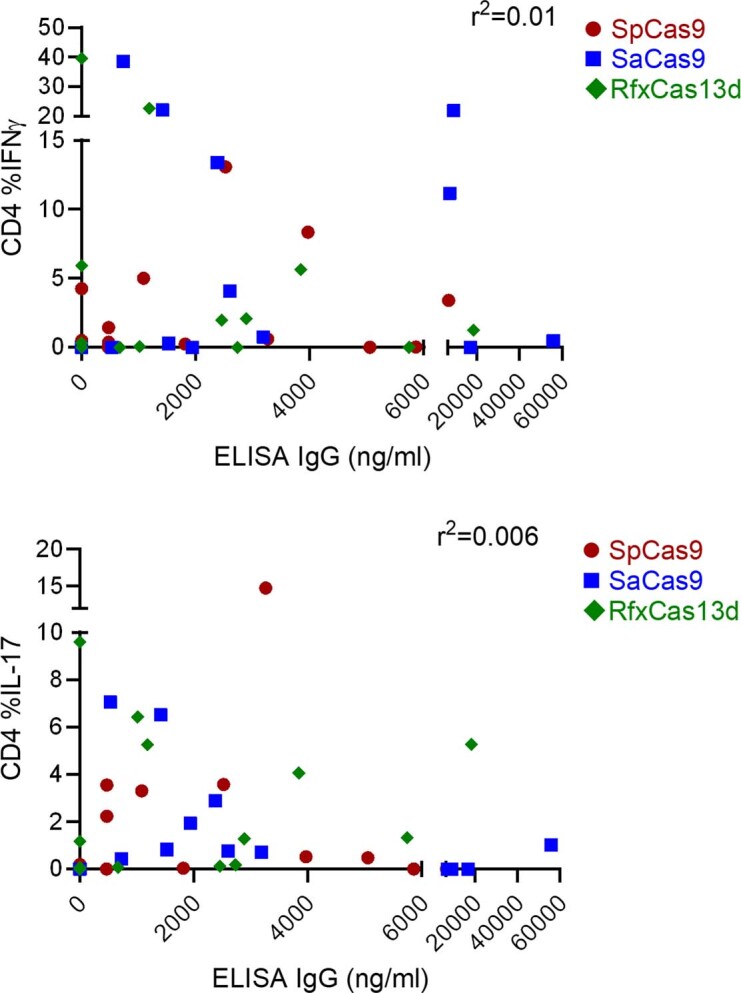
Correlation between antibody and CD4 T cell responses to recombinant Cas proteins. Frequency of CD4 T cell IFNγ (top) or IL-17 (bottom) responses were plotted against the corresponding IgG antibodies detected recognizing each of the Cas proteins in 13 samples in which paired data was available. T cell values reported are obtained after subtracting background responses from controls expanded with no antigen but re-stimulated with the tested antigen. IgG values reported are obtained after subtracting background responses from GFP controls. Source data

To further probe the cytokine potential of responding T cells, we restimulated expanded cells with autologous antigen-presenting cells (APCs) and recombinant Cas proteins on the final day of culture. Our gating strategy analyzes CD4 and CD8 T cell compartments separately, focusing on cytokine production in proliferated T cells to identify antigen-responding cells that were expanded in culture (Extended Data Fig. 2). In the CD4 T cell compartment, we observed increased IFN-γ, IL-17 and TNF-α responses to RfxCas13d, above that which was induced by GFP control (Fig. 2a,b and Extended Data Fig. 3). The cytokine responses were comparable with that induced by SpCas9 and SaCas9. We were not able to detect any T cells producing IL-10 (Extended Data Fig. 3). Further analysis of samples in which both antibody and T cell data were available (13 samples, 3 Cas proteins, 39 paired readings) found that antibody responses and T cell responses are not correlated (Extended Data Fig. 4). Overall, we noted antigen-induced inflammatory cytokine production of IFN-γ or IL-17 by the CD4 T cells in 15/24 (62%, SpCas9), 20/24 (83%, SaCas9) and 21/24 (88%, RfxCas13d) samples.

**Extended Data Fig. 5:**
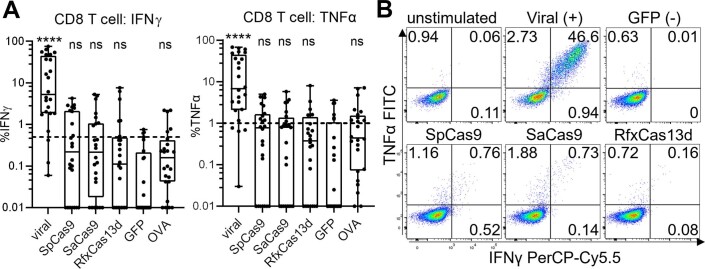
Cytokine profile of recombinant protein-stimulated RfxCas13d-specific CD8 T cells. (**a**) Aggregate responses are shown, with each data point representing one sample. (*n* = 23 biologically independent samples). Each sample was tested across all conditions shown. To ensure cytokine responses are indeed from cells expanded by antigen stimulation, each data point plots values after subtracting background responses from controls expanded with no antigen but re-stimulated with the tested antigen. Differences in treatment conditions were tested using Friedman’s test with two-sided Dunn’s multiple comparisons test using GFP as the control. (ns, not significant, * p < 0.05, ** p < 0.01, *** p < 0.001, **** p < 0.0001). For box-plots, center line indicates median, box limits indicate 25^th^ and 75^th^ percentiles, whiskers show minimum to maximum value. Dashed line indicates the limit of detection for antigen specific responses. (**b**) Representative flow cytometric analysis of cytokine responses from one sample is shown. Source data

**Extended Data Fig. 6:**
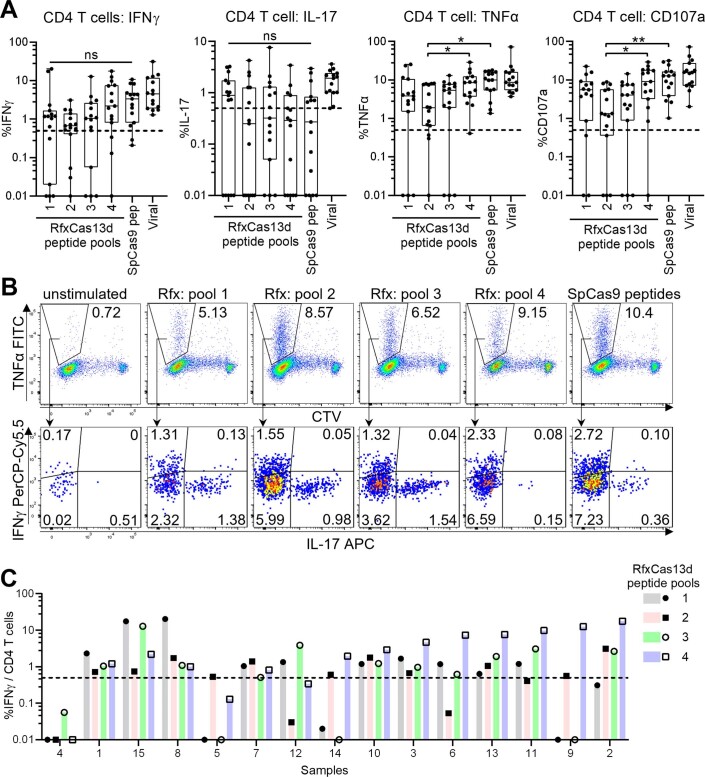
Cytokine profile of peptide-stimulated RfxCas13d specific CD4 T cells. (**a**) Aggregate responses are shown, with each data point representing one sample. (*n* = 15 biologically independent samples). Each sample was tested across all conditions shown. To ensure cytokine responses are indeed from cells expanded by peptide stimulation, each data point plots values after subtracting background responses from controls expanded with no peptide (DMSO) but re-stimulated with the tested peptide pool. For box-plots, center line indicates median, box limits indicate 25^th^ and 75^th^ percentiles, whiskers show minimum to maximum value. Dashed line indicates the limit of detection for peptide specific responses. Differences in treatment conditions were tested using Friedman’s test with two-sided Dunn’s multiple comparisons test across the SpCas9 and 4 RfxCas13d peptide pools. (ns, not significant, * p < 0.05, ** p < 0.01). (**b**) Representative flow cytometric analysis of cytokine responses from one sample is shown, indicating the detection of IFNγ and IL-17 responding cells within the TNFα producing proliferated cell population. Percentages shown are frequency among total CD4 T cells. (**c**) CD4 T cell IFNγ responses from each sample is shown. Samples are sorted based on the peptide pool which elicited the strongest responses. Source data

In the CD8 T cell compartment, we did not observe significant IFN-γ or TNF-α cytokine production higher than that induced by GFP stimulation at the population level (Extended Data Fig. 5), although we did observe IFN-γ or TNF-α cytokine levels above background in a handful of tested individuals. The whole recombinant proteins we used for restimulation are likely to be captured by the exogenous pathway for antigen presentation, to be presented predominantly on major histocompatibility complex (MHC) class II rather than MHC class I molecules, potentially leading to an underestimation of CD8 T cell responses. We proceeded to evaluate CD8 T cell responses using overlapping 15-mer peptides spanning the whole RfxCas13d protein, pooling the peptides into 4 pools. In parallel, we also stimulated PBMCs with overlapping 15-mer peptides spanning SpCas9 protein, for which others have demonstrated pre-existing CD8 T cell responses^3,11^. Our assay provided minimal stimulation for short-term expansion of memory T cells with low chance of a false positive, as demonstrated by responses below background for both GFP and ovalbumin; therefore, no negative control peptides were used. CD8 T cell responses directed against peptides from RfxCas13d were detectable, but were of considerably lower magnitude than responses directed against peptides from SpCas9. We identified IFN-γ or TNF-α cytokine responses to at least one of the four peptide pools in 11/15 (73%) samples and CD107a upregulation in 12/15 (80%) samples, indicating cytotoxic capacity (Fig. 2c,d). For SpCas9 CD8 T cell responses, 9/15 (60%) samples showed IFN-γ or TNF-α cytokine responses, and 13/15 (87%) samples showed CD107a cytotoxic responses. We also observed CD4 T cell production of IFN-γ, TNF-α and IL-17 after overlapping peptide stimulation (Extended Data Fig. 6), with all samples showing cytokine responses to at least one of the four peptide pools, confirming earlier observations from protein stimulation (Fig. 2a,b).

**Extended Data Fig. 7:**
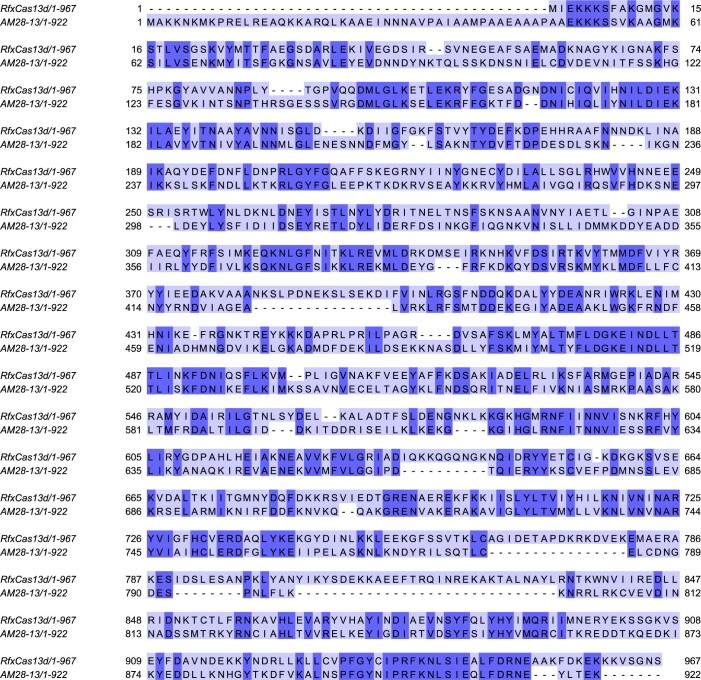
Homology between RfxCas13d and human gut isolate *Ruminoccocus sp*. AM28-13. Protein sequence alignment between RfxCas13d (top row) and human gut isolate AM28-13 (bottom row) with dark blue boxes highlighting homologous amino acids.

The detection of immune responses to RfxCas13d is contrary to expectations because *Ruminococcus flavefaciens* strain XPD3002, the bacterial origin of RfxCas13d, was originally isolated from the bovine rumen and does not colonize humans. A protein BLAST search found that the next closest protein matches were Cas13d from other bacteria belonging to *Ruminococcus* sp., such as *R. bicirculans*, a dominant human colonic bacteria^12^. These Cas13d proteins from related species in the human gut share 35–37% protein sequence identity (Extended Data Fig. 7), suggesting that T cells and antibodies that recognize regions with greater homology may be sufficiently cross-reactive to respond to RfxCas13d. To address this possibility, during peptide pooling for our peptide stimulation assay, we combined the peptides showing greater homology with human isolates in pool 4 and assigned the other peptides to pools 1–3. IFN-γ and TNF-α production by CD4 T cells was elevated in pool 4 (Extended Data Fig. 6), which also induced the highest CD4 IFN-γ responses among the 4 peptide pools in 8/15 samples, supporting the hypothesis of cross-reactive T cells. However, other peptide pools also induced specific responses, for example pool 1 appeared to be more effective at inducing IL-17 CD4 T cell responses (Extended Data Fig. 6) and pool 3 seemed to induce stronger TNF-α CD8 T cell responses (Fig. 2c). Immune responses against other Cas13 enzymes were not assessed in this study, so the generalizability of the findings is not yet clear. Detectable T cell responses elicited by peptide pools 1–3 suggest that amino acid sequences that do not have extensive homology to proteins derived from known bacteria strains that colonize humans may nonetheless elicit immune responses. Such responses can be identified only by empirical testing. Similarly, newly discovered Cas proteins derived from any source will have to be individually tested to determine whether they may be susceptible to immune recognition.

Our findings argue for caution in the use of RfxCas13d as a therapeutic, particularly if the enzyme is to be expressed in the long term. Because RfxCas13d will exert its effects on RNA in the intracellular space rather than in the extracellular space, we expect RfxCas13d to be more susceptible to T cell-mediated recognition and clearance than antibody recognition. Although most non-immune cells do not present peptides on MHC-II and may thus escape recognition by the RfxCas13d-reactive CD4 T cells, many organs and tissues contain tissue-resident macrophages that express MHC-II^13^, and may contribute to antigen presentation^14^. Our data also suggest the presence of cytotoxic CD8 T cells that recognize RfxCas13d and may play a role in eliminating cells expressing RfxCas13d.

Some have suggested the use of RfxCas13d as an anti-viral agent^15^ or to help eliminate cancerous cells in bladder cancer^16^. The immune response directed against RfxCas13d may provide an added effect in eliminating virus-infected or cancerous cells. However, IL-17-producing CD4 T cells are not the most effective T cells for killing infected or cancerous cells and may instead create a pro-inflammatory environment leading to tissue damage. In addition, precise delivery of RfxCas13d to target cells will be required so that healthy cells expressing RfxCas13d will not be vulnerable to immune-mediated toxicity.

To avoid inflammatory immune-mediated toxicity, other gene therapy strategies have adopted immunosuppressive regimens with varying degrees of success^17^. Alternatively, repeated dosing using orthologues with non-overlapping immune recognition sites may allow sustained genetic edits^18^. It may also be possible to identify immunodominant T cell epitopes and modify them to reduce immunogenicity or MHC binding affinity^11,19^, a strategy that has been used to deimmunize recombinant monoclonal antibodies facing issues of immune-mediated elimination. RfxCas13d may also be able to avoid immune detection if its therapeutic use is limited to immuno-privileged sites. Further studies will be required to determine whether these strategies may be sufficient to allow long-term expression and sustained genetic editing by RNA-directed RNA nucleases, such as RfxCas13d.

## Methods

### Human samples

Study protocols were approved by the Institutional Review Board at the National University of Singapore (H-18-072) and at Agency for Science, Technology and Research (2018-005). PBMCs and plasma were obtained from cone blood of healthy donors with informed consent at the Health Services Authority, Singapore (201906-03). Whole blood was centrifuged at 1,600*g* for 10 min at room temperature, and the clear plasma layer was extracted. The plasma layer was centrifuged at 16,000*g* for 10 min to obtain cell-free plasma supernatant. After removing the plasma layer, the cellular fraction was diluted with sterile PBS (1^st^ BASE) and layered on top of Ficoll (Ficoll-Paque, GE Healthcare), with 25 ml of diluted blood over 15 ml of Ficoll. The sample was centrifuged at 1,900*g* for 30 min at room temperature with reduced acceleration and low brakes. The PBMC layer was collected at the Ficoll-PBS interphase using a sterile Pasteur pipette and washed extensively with PBS. PBMCs were cryopreserved in CryoStor CS10 freezing medium (Stemcell Technologies) or freezing medium containing 90% FBS (Hyclone) and 10% DMSO (Hybri-Max D2650, Sigma).

### Recombinant Cas proteins

For recombinant Cas protein or GFP expression, bacteria-optimized cas protein genes were synthesized (IDT) and cloned into a pET28 backbone with N-terminal 6×His-maltose-binding protein (MBP)-TEV protease cleavage site for expression from T7 promoter. His-MBP-Cas expression plasmids were sent for protein production by the Protein Production Platform (PPP) at Nanyang Technological University School of Biological Sciences (www.proteins.sbs.ntu.edu.sg). GFP was previously cloned by PPP into the pNIC28-Bsa4 backbone encoding an amino-terminal 6×His-TEV protease site. Plasmids were transformed into *Escherichia coli* BL21(DE3) Rosetta, and expression cultures were grown at 37 °C using the LEX Bioreactor (Harbinger Biotech) in Terrific Broth medium supplemented with appropriate antibiotics. Upon reaching an OD_600_ of 2.0, expression cultures were cooled down to 18 °C before overexpression was induced by adding 0.5 mM IPTG. Cell pellets were collected at 20 hours after induction, resuspended in lysis buffer (100 mM HEPES, 500 mM NaCl, 10 mM imidazole, 10% glycerol, 0.5 mM TCEP, pH 8.0, 0.1% Nacalai Tesque protease inhibitor cocktail) and sonicated. After clarification by centrifugation at 47,000*g*, lysate supernatants were filtered and loaded onto an AKTAxpress system (GE Healthcare) for ion-metal affinity chromatography, followed by size-exclusion chromatography. All Ni-NTA columns (Qiagen) were washed with buffers containing 10 mM and 25 mM imidazole before proteins were eluted with elution buffer (20 mM HEPES, 500 mM NaCl, 500 mM Imidazole, 10% (vol/vol) glycerol, 0.5 mM TCEP, pH 7.5). The proteins were then subjected to size-exclusion chromatography in gel filtration buffer (20 mM HEPES, 300 mM NaCl, 10% (vol/vol) glycerol, 0.5 mM TCEP, pH 7.5) on a HiLoad 16/60 Superdex 75 prep grade column (for GFP) or Superdex 200 (for MBP-Cas). Fractions containing pure protein were pooled, supplemented with TCEP up to 2.0 mM and concentrated using Vivaspin 20 concentrators (Cytiva) before being subjected to overnight TEV protease treatment at 4 °C. Samples were then loaded onto Ni-NTA columns again so we could collect the untagged proteins. Protein purity was determined to be >90% by SDS–PAGE (Supplementary Fig. 1). Endotoxins were removed using High Capacity Endotoxin Removal spin columns (Pierce, Thermo Fisher) and quantified using LAL Chromogenic Endotoxin Quantitation Kit (Pierce, Thermo Fisher). All protein preparations had less than 0.2 EU/ml of endotoxins when tested at a final concentration of 10 µg/ml. Residual *E. coli* host cell proteins were present at less than 2% in all protein preparations when tested using an *E. coli* host cell protein ELISA kit (Abcam).

### ELISA

A direct ELISA assay was developed to detect IgG antibodies specific to Cas proteins. Ninety-six-well Nunc Maxisorp plates (Thermo Fisher) were coated with Cas proteins or GFP at 2 µg/ml in 100 µl of PBS (1^st^BASE) for 2–3 h at 37 °C. For standards, wells were coated with twofold serial dilutions of purified human IgG (I4506, Sigma-Aldrich) from 320 ng/ml to 5 ng/ml. The wells were washed with Wash Buffer (PBS, 0.05% Tween 20 (Vivantis Technologies)) and blocked with 250 µl Blocking Buffer (PBS, 0.1% Tween 20 and 1% Bovine Serum Albumin (BSA, Fraction V, Gold Biotechnology)) overnight at 4 °C. After 2 washes with Wash buffer, diluted plasma samples were added to the wells and incubated at room temperature for 2 hours. Plasma samples were diluted with Assay Buffer (PBS, 0.05% Tween 20 and 0.5% BSA) and tested at final concentrations of 1:20, 1:40, 1:80 and 1:160 dilutions. Readings within the linear range of standards were used to calculate IgG concentration. After extensive washing, monoclonal mouse anti-human IgG HRP (Invitrogen, Life Technologies) was used to detect bound antigen-specific IgG. After unbound antibodies were washed off, TMB Substrate solution (eBioscience, Life Technologies) was added to wells for 20 min at room temperature and Stop Solution (Invitrogen, Life Technologies) was added to halt the reaction. Absorbance was measured at 450 nm with wavelength subtraction at 570 nm on the microplate reader (Synergy HTX, Biotek Instruments) using Gen5 software (Biotek Instruments).

### T cell cultures

PBMCs were rapidly thawed at 37 °C and washed with a tenfold volume of RPMI (Hyclone) or DMEM (Hyclone). To measure proliferation in 10-day cultures, cells were uniformly labeled with CellTrace Violet (CTV, Life Technologies) as described^20^. Briefly, PBMCs were resuspended in PBS (1^st^ BASE) with 2% AB serum (H6914, Sigma-Aldrich) and mixed in a 1:1 ratio with diluted CTV at a final concentration of 5 µM. PBMCs were incubated in a 37 °C water bath for 20 minutes, with gentle mixing every 5–10 minutes. PBMCs were washed extensively and resuspended in AIM-V medium (Gibco, Thermo Fisher) with 2% AB serum. Then, 2–3 × 10^5^ cells were plated per well in a 96-well round-bottom plate (Corning) in the presence of 20 IU/ml of recombinant human IL-2 (Stemcell Technologies), as has been described^21^. PBMCs were left either unstimulated with no additional antigen or were stimulated with 5 µg/ml of recombinant Cas proteins or GFP. As an additional negative control for comparison with GFP, cells were also cultured with low-endotoxin ovalbumin (LS003061, Worthington). As a positive control, PBMCs were stimulated with 1 µg/ml of a mix of viral peptides comprising CEF peptides (Mabtech and Proimmune) to activate CD8 T cells and CEFT peptides (Proimmune) to activate CD4 T cells. For cultures stimulated with peptides, 15-mer peptides overlapping by 10 amino acids spanning the whole RfxCas13d protein was custom ordered from Genscript and pooled. Each peptide is assigned to one of four pools. The peptides present in each pool are indicated in Supplementary Table 1. Overlapping peptides for SpCas9 Pepmix was from JPT Peptide Technologies. PBMCs were stimulated with 2.5 µg/ml of each peptide with the final concentration of DMSO at less than 0.5%. For peptide-stimulated cultures, corresponding negative control wells were stimulated with 0.5% DMSO. Negative control peptides were not used with peptide-stimulated cultures because the short-term T cell expansion protocol uses minimal stimulants with low dose IL-2, synthetic peptides and no additional cytokines, co-stimulatory signals or antigen-presenting cells, thus preferentially favoring the proliferation of antigen-experienced cells over any naive cells. Recent studies using similar T cell expansion assays to evaluate antigen-specific T cell responses also do not use negative control peptides^22,23^. Cultures were split and IL-2 medium was replenished as needed on days 4, 7 and 10 of culture.

To determine the cytokine potential of activated T cells, autologous antigen-presenting cells (APCs) were prepared for T cell restimulation on day 10. Thawed PBMCs were labeled with anti-CD2-biotin antibodies (Biolegend) in cold MACS buffer (PBS, 0.5% BSA, 2 mM EDTA), and CD2-positive T cells and natural killer cells were depleted using Streptavidin MyOne T1 Dynabeads (Life Technologies). The CD2-negative fraction comprising predominantly blood monocytes was cultured in 96-well round-bottom plates at 1 × 10^5^–1.5 × 10^5^ cells/well in the presence of recombinant Cas proteins, GFP, ovalbumin (5 µg/ml) or peptides from RfxCas13d, SpCas9 or viral peptide pools, as above for antigen capture. After 16–18 hours, expanded T cells were added to the APCs for restimulation in the presence of anti-CD28 antibody (1 µg/ml, LEAF-purified, Biolegend). To assess cytokine production, brefeldin A (10 µg/ml, eBioscience, Life Technologies) and monensin (2 µM, eBioscience, Life Technologies) were added to cultures 4–6 hours before staining for flow cytometric analysis. For peptide expansion and stimulation experiments, anti-CD107a PE antibody (Biolegend) was added into culture for 1 hour at 37 °C before brefeldin a and monensin were added. For positive controls, T cells expanded with viral peptides were re-stimulated by adding to APCs cultured in the presence of viral peptides. Some cells expanded using viral peptides were separately stimulated with phorbol 12-myristate 13-acetate (PMA) and ionomycin (Cell Stimulation Cocktail, eBioscience, Life Technologies) as flow-cytometry controls. To ensure that cytokine-producing cells were indeed expanded by 10 day cultures, PBMCs cultured for 10 days without antigen were also restimulated with APCs that had captured protein or peptide antigen in the presence of anti-CD28 antibody to determine baseline non-antigen-specific cytokine production. Any detected responses were subtracted as background. All donors showed increased proliferation and cytokine responses in T cells after Cas9 or Cas13 stimulation compared with GFP stimulation, demonstrating that we could detect Cas9- and Cas13-specific responses over and above any background residual host protein responses in all donors, thus supporting the inclusion of all donors for the T cell analysis.

### Flow cytometry

Cultured cells were washed twice with FACS buffer (PBS, 2% FBS, 0.1% BSA, 2 mM EDTA, 0.01% sodium azide (S2002, Sigma-Aldrich)) and stained for flow cytometry, as has been described^20^. Cells were incubated with Human Fc Block (BD) for 10–15 minutes on ice before staining antibodies were added. Antibodies used are listed in Supplementary Table 2. Cells were incubated with antibody mixes for 20–30 minutes on ice and washed twice before acquisition. For intracellular cytokine staining, cells were fixed and permeabilized with Cytofix/Cytoperm solution (BD) for 20 minutes on ice, then washed with Perm/Wash buffer (BD). Cells were stained with intracellular staining antibodies in Perm/Wash Buffer for 20 minutes on ice, then washed twice before acquisition. Cells were acquired on LSR Fortessa or X-20 (BD) using FACS DiVa software (Version 9, BD) and analyzed on FlowJo software (Version 10.8, BD).

### Statistical analyses

ELISA data was analyzed using GainData (Arigo Biolaboratories, https://www.arigobio.com/elisa-analysis). Standards were plotted using 5 parameter logistic regression. Plasma was tested at 4 dilutions (1:20, 1:40, 1:80, 1:160) and readings were obtained within the linear region of the standard curve were used to calculate antigen-specific IgG concentrations. For all other data, GraphPad Prism 9 (Graphpad Software) was used for statistical analysis and graphical representations. The appropriate statistical tests were chosen on the basis of experimental design after consulting the GraphPad Statistics Guide.

### Homology analysis

Protein sequence for RfxCas13d (accession number: SFX39573.1) was aligned with one of the top hits found using NCBI Protein Blast, Cas13d from a human gut isolate *Ruminococcus sp*. AM28-13 (accession number: WP_117925375.1). Alignment was completed using Clustal W (1.83) on EBI tools and alignment figure was generated in Jalview^24^.

### Reporting summary

Further information on research design is available in the Nature Research Reporting summary linked to this article.

## Online content

Any methods, additional references, Nature Research reporting summaries, source data, extended data, supplementary information, acknowledgements, peer review information; details of author contributions and competing interests; and statements of data and code availability are available at 10.1038/s41591-022-01848-6.

## Supplementary information

Supplementary Information Supplementary Figure 1 and Supplementary Tables 1–3

Reporting Summary

## Data Availability

Source data are available for Figs. 1 and 2 and Extended Data Figs. 1, 3, and 4–6. The raw SDS–PAGE gel images for the purified Cas enzymes and GFP are provided in Supplementary Fig. 1. Flow cytometry data has been deposited in Flow Repository. (http://flowrepository.org/id/RvFrbjuBY5uDR7bGwyHHFMjY5JhNlSrtMZ5dqhdPHz8MyoB5J91O8bhzvSaNMlRy and http://flowrepository.org/id/RvFrmivRKvtimNgsuJBRON7bHflEHGIS4HJvikD0t5FWuuVU8Cscfj00je6LkWbD) Source data are provided with this paper.

## Source data

Source Data Fig. 1 Statistical source data
Source Data Fig. 2 Statistical source data
Source Data Extended Data Fig. 1 Statistical source data
Source Data Extended Data Fig. 3 Statistical source data
Source Data Extended Data Fig. 4 Statistical source data
Source Data Extended Data Fig. 5 Statistical source data
Source Data Extended Data Fig. 6 Statistical source data

## Code availability

No custom codes were used in this study.
