# Supplementary material for: Pre-existing adaptive immunity to the RNA-editing enzyme Cas13d in humans
Source: Nat Med. 2022 Jun 6;28(7):1372–6. doi: 10.1038/s41591-022-01848-6 (PMC9307479; doi:10.1038/s41591-022-01848-6)
Supplement: Supplementary file 1 — Supplementary Figure 1 and Supplementary Tables 1–3 [file 41591_2022_1848_MOESM1_ESM.pdf]

nature portfolio

https://doi.org/10.1038/s41591-022-01848-6

Supplementary information

Pre-existing adaptive immunity to the RNA-editing enzyme Cas13d in humans

In the format provided by the authors and unedited

Supplementary Information

Supplementary Figure 1

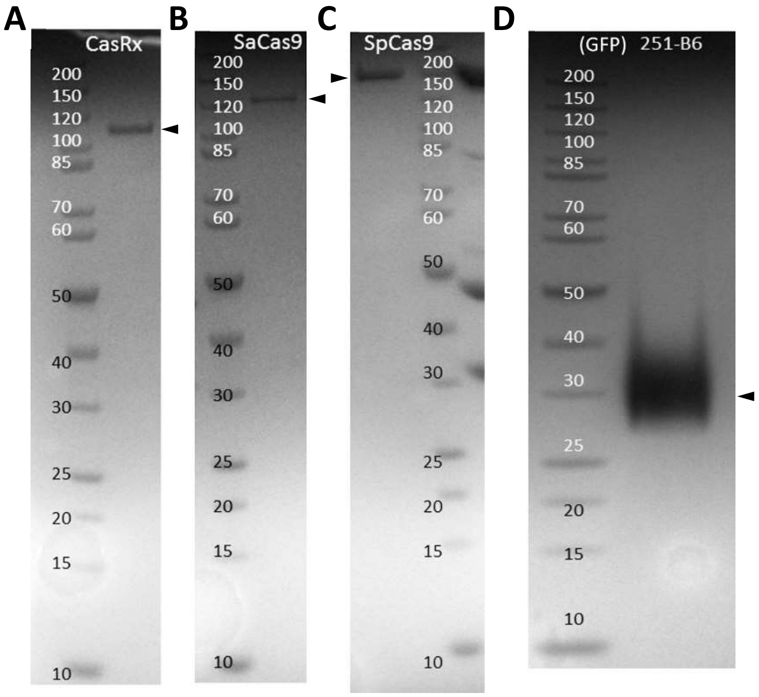

Original SDS-PAGE gel images of purified RfxCas13d (A, denoted as CasRx), SaCas9 (B), SpCas9 (C), and GFP (D) proteins obtained from the protein production facility at Nanyang Technological University.

Supplementary Table 1: Table of Rfxcas13d Overlapping Peptides in Each Pool

Pool 1	Pool 2	Pool 3	Pool 4
IEKKKSFAKGMGVKS	LREVMLDRKDMSEIR	KQGQNGKNQIDRYYE	LYTGPVQQDMLGLKE
SFAKGMGVKSTLVSG	LDRKDMSEIRKNHKV	GKNQIDRYYETCIGK	VQQDMLGLKETLEKR
MGVKSTLVSGSKVYM	MSEIRKNHKVFDSIR	DRYYETCIGKDKGKS	LGLKETLEKRYFGES
TLVSGSKVYMTTFAE	KNHKVFDSIRTKVYT	TCIGKDKGKSVSEKV	TLEKRYFGESADGND
SKVYMTTFAEGSDAR	TKVYTMMDFVIYRYY	DKGKSVSEKVDALTK	YFGESADGNDNICIQ
TTFAEGSDARLEKIV	MMDFVIYRYYIEEDA	VSEKVDALTKIITGM	ADGNDNICIQVIHNI
GSDARLEKIVEGDSI	IYRYYIEEDAKVAAA	DALTKIITGMNYDQF	NICIQVIHNILDIEK
LEKIVEGDSIRSVNE	IEEDAKVAAANKSLP	IITGMNYDQFDKKRS	VIHNILDIEKILAEY
EGDSIRSVNEGEAFS	KVAAANKSLPDNEKS	NYDQFDKKRSVIEDT	LDIEKILAEYITNAA
RSVNEGEAFSAEMAD	NKSLPDNEKSLSEKD	DKKRSVIEDTGRENA	ILAEYITNAAYAVNN
GEAFSAEMADKNAGY	DNEKSLSEKDIFVIN	VIEDTGRENAEREKF	ITNAAYAVNNISGLD
AEMADKNAGYKIGNA	LSEKDIFVINLRGSF	AQLYKEKGYDINLKK	DNFLDNPRLGYFGQA
KNAGYKIGNAKFSHP	IWRKLENIMHNIKEF	EKGYDINLKKLEEKG	NPRLGYFGQAFFSKE
KIGNAKFSHPKGYAV	ENIMHNIKEFRGNKT	YDINLKKLEEKGFSS	SIMKEQKNLGFNITK
KFSHPKGYAVVANNP	NIKEFRGNKTREYKK	LEEKGFSSVTKLCAG	QKNLGFNITKLREVM
KGYAVVANNPLYTGP	RGNKTREYKKKDAPR	FSSVTKLCAGIDETA	FNITKLREVMLDRKD
VANNPLYTGPVQQDM	REYKKKDAPRLPRIL	KLCAGIDETAPDKRK	FDSIRTKVYTMMDFV
YAVNNISGLDKDIIG	KDAPRLPRILPAGRD	IDETAPDKRKDVEKE	IFVINLRGSFNDDQK
ISGLDKDIIGFGKFS	LPRILPAGRDVSAFS	PDKRKDVEKEMAERA	LRGSFNDDQKDALYY
KDIIGFGKFSTVYTY	PAGRDVSAFSKLMYA	DVEKEMAERAKESID	NDDQKDALYYDEANR
FGKFSTVYTYDEFKD	SFLKVMPLIGVNAKF	MAERAKESIDSLESA	DALYYDEANRIWRKL
TVYTYDEFKDPEHHR	MPLIGVNAKFVEEYA	KESIDSLESANPKLY	DEANRIWRKLENIMH
DEFKDPEHHRAAFNN	VNAKFVEEYAFFKDS	SLESANPKLYANYIK	VSAFSKLMYALTMFL
PEHHRAAFNNNDKLI	VEEYAFFKDSAKIAD	NPKLYANYIKYSDEK	KLMYALTMFLDGKEI
AAFNNNDKLINAIKA	FFKDSAKIADELRLI	ANYIKYSDEKKAEEF	LTMFLDGKEINDLLT
NDKLINAIKAQYDEF	AKIADELRLIKSFAR	YSDEKKAEEFTRQIN	DGKEINDLLTTLINK
NAIKAQYDEFDNFLD	ELRLIKSFARMGEPI	KAEEFTRQINREKAK	NDLLTTLINKFDNIQ
QYDEFDNFLDNPRLG	KSFARMGEPIADARR	TRQINREKAKTALNA	TLINKFDNIQSFLKV
YFGQAFFSKEGRNYI	MGEPIADARRAMYID	REKAKTALNAYLRNT	FDNIQSFLKVMPLIG
FFSKEGRNYIINYGN	ADARRAMYIDAIRIL	TALNAYLRNTKWNVI	KKGKHGMRNFIINNV
GRNYIINYGNECYDI	AMYIDAIRILGTNLS	YLRNTKWNVIIREDL	GMRNFIINNVISNKR
INYGNECYDILALLS	AIRILGTNLSYDELK	KWNVIIREDLLRIDN	IINNVISNKRFHYLI
ECYDILALLSGLRHW	GTNLSYDELKALADT	IREDLLRIDNKTCTL	GRENAEREKFKKIIS
LALLSGLRHWVVHNN	YDELKALADTFSLDE	LRIDNKTCTLFRNKA	EREKFKKIISLYLTV
GLRHWVVHNNEEESR	ALADTFSLDENGNKL	KTCTLFRNKAVHLEV	KKIISLYLTVIYHIL
VVHNNEEESRISRTW	FSLDENGNKLKKGKH	FRNKAVHLEVARYVH	LYLTVIYHILKNIVN
EEESRISRTWLYNLD	NGNKLKKGKHGMRNF	VHLEVARYVHAYIND	IYHILKNIVNINARY
ISRTWLYNLDKNLDN	ISNKRFHYLIRYGDP	QRIIMNERYEKSSGK	KNIVNINARYVIGFH
LYNLDKNLDNEYIST	FHYLIRYGDPAHLHE	NERYEKSSGKVSEYF	INARYVIGFHCVERD
KNLDNEYISTLNYLY	RYGDPAHLHEIAKNE	KSSGKVSEYFDAVND	VIGFHCVERDAQLYK
EYISTLNYLYDRITN	AHLHEIAKNEAVVKF	VSEYFDAVNDEKKYN	CVERDAQLYKEKGYD
LNYLYDRITNELTNS	IAKNEAVVKFVLGRI	DAVNDEKKYNDRLLK	ARYVHAYINDIAEVN
DRITNELTNSFSKNS	AVVKFVLGRIADIQK	EKKYNDRLLKLLCVP	IAEVNSYFQLYHYIM
ELTNSFSKNSAANVN	VLGRIADIQKKQGQN	EAAKFDKEKKKVSGN	SYFQLYHYIMQRIIM
FSKNSAANVNYIAET	ADIQKKQGQNGKNQI	DKEKKKVSGNS	YHYIMQRIIMNERYE
AANVNYIAETLGINP			DRLLKLLCVPFGYCI
YIAETLGINPAEFAE			LLCVPFGYCIPRFKN
LGINPAEFAEQYFRF			FGYCIPRFKNLSIEA
AEFAEQYFRFSIMKE			PRFKNLSIEALFDRN
QYFRFSIMKEQKNLG			LSIEALFDRNEAAKF
			LFDRNEAAKFDKEKK

Supplementary Table 2: Table of Antibodies Used.

For T cell culture	Concentration	Clone	Supplier, Catalog No.	Validation
LEAF anti-CD28	1μg/ml	CD28.2	Biolegend 302933	https://www.biolegend.com/en-us/products/ultra-leaf-purified-anti-human-cd28-antibody-7743
Biotin anti-CD2	5μg/ml	RPA-2.10	Biolegend 300204	https://www.biolegend.com/en-us/products/biotin-anti-human-cd2-antibody-817

For flow cytometry	Dilution	Clone	Supplier	
Human Fc Block	1:50	Fc1.3216	BD 564220	https://www.bdbiosciences.com/content/bdb/paths/generate-tds-document.eu.564219.pdf
CD3 Super Bright 600	1:50	OKT3	eBioscience 63-0037-42	https://www.thermofisher.com/antibody/product/CD3-Antibody-clone-OKT3-Monoclonal/63-0037-42
CD4 Alexa Fluor 700	1:50	SK3	Biolegend 344622	https://www.biolegend.com/en-us/products/alexa-fluor-700-anti-human-cd4-antibody-9354
CD4 Alexa Fluor 700	1:50	SK3	eBioscience 56-0047-42	https://www.thermofisher.com/antibody/product/CD4-Antibody-clone-SK3-SK-3-Monoclonal/56-0047-42
CD8 Brilliant Violet 785	1:50	SK1	Biolegend 344740	https://www.biolegend.com/en-us/products/brilliant-violet-785-anti-human-cd8-antibody-12175
TNFα FITC	1:50	MAb11	Biolegend 502906	https://www.biolegend.com/en-us/products/fitc-anti-human-tnf-alpha-antibody-1345
IFNγ PerCP-Cy5.5	1:50	4S.B3	Biolegend 502526	https://www.biolegend.com/en-us/products/percp-cyanine5-5-anti-human-ifn-gamma-antibody-4426
IL-17 APC	1:50	BL168	Biolegend 512334	https://www.biolegend.com/en-us/products/apc-anti-human-il-17a-antibody-9987
IL-10 PE	2:50	JES3-9D7	Biolegend 501404	https://www.biolegend.com/en-us/products/pe-anti-human-il-10-antibody-1341
CD107a PE	1:2000	H4A3	Biolegend 328608	https://www.biolegend.com/en-us/products/pe-anti-human-cd107a-lamp-1-antibody-4967
CD3 PE-Cy7	1:50	SK7	Biolegend 344816	https://www.biolegend.com/en-us/products/pe-cyanine7-anti-human-cd3-antibody-6934
Fixable Viability Dye eFluor780	1:600		eBioscience 65-0865-14	https://www.thermofisher.com/order/catalog/product/65-0865-14

Supplementary Table 3: Table of exact p values

Figure 1A	Adjusted p value
gfp vs. spcas9	<1.000000000e-004
gfp vs. sacas9	<1.000000000e-004
gfp vs. casrx	<1.000000000e-004

Figure 1C	Adjusted p value
baseline vs. viral	0.179751563
baseline vs. SpCas9	0.000126936
baseline vs. SaCas9	0.000108105
baseline vs. RfxCas13d	<1.000000000e-004
baseline vs. GFP	0.23845564
baseline vs. OVA	0.407245117

Figure 1D	Adjusted p value
baseline vs. viral	<1.000000000e-004
baseline vs. SpCas9	<1.000000000e-004
baseline vs. SaCas9	<1.000000000e-004
baseline vs. RfxCas13d	<1.000000000e-004
baseline vs. GFP	0.999564374
baseline vs. OVA	0.167669019

Figure 2A IFNg (left)	Adjusted p value
GFP vs. viral	8.8385E-09
GFP vs. SpCas9	0.008901053
GFP vs. SaCas9	0.000782782
GFP vs. RfxCas13d	0.00143851
GFP vs. OVA	>9.999999999e-001

Figure 2A IL-17 (right)	Adjusted p value
GFP vs. viral	6.53083E-05
GFP vs. SpCas9	0.000572567
GFP vs. SaCas9	4.81306E-06
GFP vs. RfxCas13d	2.06167E-09
GFP vs. OVA	0.448164971

Figure 2C IFNg (left)	Adjusted p value
1 vs. 2	>9.999999999e-001
1 vs. 3	>9.999999999e-001
1 vs. 4	>9.999999999e-001
1 vs. SpCas9 pep	0.433081428
2 vs. 3	>9.999999999e-001
2 vs. 4	>9.999999999e-001
2 vs. SpCas9 pep	0.567468165
3 vs. 4	>9.999999999e-001
3 vs. SpCas9 pep	>9.999999999e-001
4 vs. SpCas9 pep	0.940685547

Figure 2C TNFa (middle)	Adjusted p value
1 vs. 2	>9.999999999e-001
1 vs. 3	>9.999999999e-001
1 vs. 4	>9.999999999e-001
1 vs. SpCas9 pep	0.079117887
2 vs. 3	0.179263391
2 vs. 4	>9.999999999e-001
2 vs. SpCas9 pep	0.002198507
3 vs. 4	>9.999999999e-001
3 vs. SpCas9 pep	>9.999999999e-001
4 vs. SpCas9 pep	0.153138217

Figure 2C CD107a (right)	Adjusted p value
1 vs. 2	>9.999999999e-001
1 vs. 3	>9.999999999e-001
1 vs. 4	>9.999999999e-001
1 vs. SpCas9 pep	0.046691318
2 vs. 3	>9.999999999e-001
2 vs. 4	>9.999999999e-001
2 vs. SpCas9 pep	0.026801717
3 vs. 4	>9.999999999e-001
3 vs. SpCas9 pep	0.110744377
4 vs. SpCas9 pep	0.093747685

Ext Data Fig 3A	Adjusted p value
GFP vs. Viral	9.26446E-05
GFP vs. SpCas9	0.021544719
GFP vs. SaCas9	6.53083E-05
GFP vs. RfxCas13d	0.001933654
GFP vs. OVA	>9.999999999e-001

Ext Data Fig 3C	Adjusted p value
GFP vs. Viral	>9.999999999e-001
GFP vs. SpCas9	>9.999999999e-001
GFP vs. SaCas9	>9.999999999e-001
GFP vs. RfxCas13d	>9.999999999e-001
GFP vs. OVA	>9.999999999e-001

Ext Data Fig 5A IFNg (left)	Adjusted p value
GFP vs. viral	2.85646E-08
GFP vs. SpCas9	0.103187791
GFP vs. SaCas9	0.153767806

GFP vs. RfxCas13d	>9.999999999e-001
GFP vs. OVA	0.662317834

Ext Data Fig 5A TNFa (right)	Adjusted p value
GFP vs. viral	1.11271E-07
GFP vs. SpCas9	0.139452646
GFP vs. SaCas9	0.093081602
GFP vs. RfxCas13d	0.412896371
GFP vs. OVA	0.126301508

Ext Data Fig 6A IFNg	Adjusted p value
1 vs. 2	>9.999999999e-001
1 vs. 3	>9.999999999e-001
1 vs. 4	>9.999999999e-001
1 vs. SpCas9 pep	>9.999999999e-001
2 vs. 3	>9.999999999e-001
2 vs. 4	0.282403687
2 vs. SpCas9 pep	0.153138217
3 vs. 4	0.646716875
3 vs. SpCas9 pep	0.376669222
4 vs. SpCas9 pep	>9.999999999e-001

Ext Data Fig 6A IL-17	Adjusted p value
1 vs. 2	>9.999999999e-001
1 vs. 3	>9.999999999e-001
1 vs. 4	>9.999999999e-001
1 vs. SpCas9 pep	>9.999999999e-001
2 vs. 3	>9.999999999e-001
2 vs. 4	>9.999999999e-001
2 vs. SpCas9 pep	>9.999999999e-001
3 vs. 4	>9.999999999e-001
3 vs. SpCas9 pep	>9.999999999e-001
4 vs. SpCas9 pep	>9.999999999e-001

Ext Data Fig 6A TNFa	Adjusted p value
1 vs. 2	>9.999999999e-001
1 vs. 3	>9.999999999e-001
1 vs. 4	0.282403687
1 vs. SpCas9 pep	0.376669222
2 vs. 3	>9.999999999e-001
2 vs. 4	0.018227352
2 vs. SpCas9 pep	0.026801717
3 vs. 4	0.376669222
3 vs. SpCas9 pep	0.496472297
4 vs. SpCas9 pep	>9.999999999e-001

Ext Data Fig 6A CD107a	Adjusted p value
1 vs. 2	>9.999999999e-001
1 vs. 3	>9.999999999e-001
1 vs. 4	0.832645167
1 vs. SpCas9 pep	0.326634417
2 vs. 3	>9.999999999e-001
2 vs. 4	0.032349119
2 vs. SpCas9 pep	0.008121171
3 vs. 4	0.179263391
3 vs. SpCas9 pep	0.055836168
4 vs. SpCas9 pep	>9.999999999e-001
