# Supplementary material for: Pre-existing adaptive immunity to the RNA-editing enzyme Cas13d in humans
Source: Nat Med. 2022 Jun 6;28(7):1372–6. doi: 10.1038/s41591-022-01848-6 (PMC9307479; doi:10.1038/s41591-022-01848-6)
Supplement: Supplementary file 2 — Reporting Summary [file 41591_2022_1848_MOESM2_ESM.pdf]

Corresponding author(s): Gene Yeo

Last updated by author(s): Apr 12, 2022

Reporting Summary

Nature Portfolio wishes to improve the reproducibility of the work that we publish. This form provides structure for consistency and transparency in reporting. For further information on Nature Portfolio policies, see our Editorial Policies and the Editorial Policy Checklist.

Statistics

For all statistical analyses, confirm that the following items are present in the figure legend, table legend, main text, or Methods section.

n/a	Confirmed	
☐	☒	The exact sample size (*n*) for each experimental group/condition, given as a discrete number and unit of measurement
☐	☒	A statement on whether measurements were taken from distinct samples or whether the same sample was measured repeatedly
☐	☒	The statistical test(s) used AND whether they are one- or two-sided<br>*Only common tests should be described solely by name; describe more complex techniques in the Methods section.*
☒	☐	A description of all covariates tested
☐	☒	A description of any assumptions or corrections, such as tests of normality and adjustment for multiple comparisons
☒	☐	A full description of the statistical parameters including central tendency (e.g. means) or other basic estimates (e.g. regression coefficient) AND variation (e.g. standard deviation) or associated estimates of uncertainty (e.g. confidence intervals)
☐	☒	For null hypothesis testing, the test statistic (e.g. *F*, *t*, *r*) with confidence intervals, effect sizes, degrees of freedom and *P* value noted<br>*Give P values as exact values whenever suitable.*
☒	☐	For Bayesian analysis, information on the choice of priors and Markov chain Monte Carlo settings
☒	☐	For hierarchical and complex designs, identification of the appropriate level for tests and full reporting of outcomes
☒	☐	Estimates of effect sizes (e.g. Cohen's *d*, Pearson's *r*), indicating how they were calculated

Our web collection on statistics for biologists contains articles on many of the points above.

Software and code

Policy information about availability of computer code

Data collection	Flow cytometric data was collected using FACS DiVa (BD) software. No custom code was used.
Data analysis	ELISA data was analyzed using GainData (Arigo Biolaboratories). Flow cytometric data was analysed using FlowJo (V10.8, BD). Statistical analysis and graphical representations were done using Prism (V9, GraphPad). Protein sequence for RfxCas13d (Accession Number: SFX39573.1) was aligned with one of the top hits found using NCBI Protein Blast, Cas13d from a human gut isolate Ruminococcus sp. AM28-13 (Accession Number: WP_117925375.1). Alignment was completed using Clustal W (1.83) on EBI tools and alignment figure was generated in Jalview. No custom code was used.

For manuscripts utilizing custom algorithms or software that are central to the research but not yet described in published literature, software must be made available to editors and reviewers. We strongly encourage code deposition in a community repository (e.g. GitHub). See the Nature Portfolio guidelines for submitting code & software for further information.

Data

Policy information about availability of data

All manuscripts must include a data availability statement. This statement should provide the following information, where applicable:

- Accession codes, unique identifiers, or web links for publicly available datasets
- A description of any restrictions on data availability
- For clinical datasets or third party data, please ensure that the statement adheres to our policy

Source data are available for Figures 1 and 2 and Extended Data Figures 1, 3, 4, 5, 6. The raw SDS-PAGE gel images for the purified Cas enzymes and GFP are provided in Supplementary Figure 1. Flow cytometry data has been deposited in Flow Repository. (http://flowrepository.org/id/

RvFrbjuBY5uDR7bGwyHHFMjY5JhNlSrtMZ5dqhdPHz8MyoB5J91O8bhzvSaNMlRy and http://flowrepository.org/id/RvFrmivRKvtimNgsuJBRON7bHflEHGIS4HJvikD0t5FWuuVU8Cscfj00je6LkWbD)

Field-specific reporting

Please select the one below that is the best fit for your research. If you are not sure, read the appropriate sections before making your selection.

☒ Life sciences ☐ Behavioural & social sciences ☐ Ecological, evolutionary & environmental sciences

For a reference copy of the document with all sections, see nature.com/documents/nr-reporting-summary-flat.pdf

Life sciences study design

All studies must disclose on these points even when the disclosure is negative.

Sample size	We used a sample size of 15-24 samples. No sample size calculation was performed. This study intends to obtain a general idea of whether the healthy human population possess pre-existing immunity recognizing the antigens tested and does not intend to define exact prevalence or confidence intervals. We analysed samples as they were collected for the ELISA and protein stimulation assays, and stopped when additional samples did not alter trends observed. From the protein stimulation assays, we found that 15 samples were sufficient to establish trends, therefore 15 samples were analysed for the peptide stimulation assays.
Data exclusions	For ELISA, data were excluded if responses to GFP were unusually high, indicating that there may be other substances within the protein preparation or plasma sample that may be non-specifically inducing HRP activity. In such situations, any responses observed could not be reliably attributed to the specific protein antigen. For flow cytometry, no data were excluded.
Replication	The assay was repeated in 3 individual samples and all 3 samples showed similar results upon replication, ensuring that the results are reproducible.
Randomization	There are no experimental groups, all samples were assayed the same way.
Blinding	Investigators are blind to all donor demographic information. All samples are assayed the same way with no separation into experimental groups.

Reporting for specific materials, systems and methods

We require information from authors about some types of materials, experimental systems and methods used in many studies. Here, indicate whether each material, system or method listed is relevant to your study. If you are not sure if a list item applies to your research, read the appropriate section before selecting a response.

Materials & experimental systems

n/a	Involved in the study	
☐	☒	Antibodies
☒	☐	Eukaryotic cell lines
☒	☐	Palaeontology and archaeology
☒	☐	Animals and other organisms
☐	☒	Human research participants
☒	☐	Clinical data
☒	☐	Dual use research of concern

Methods

n/a	Involved in the study	
☒	☐	ChIP-seq
☐	☒	Flow cytometry
☒	☐	MRI-based neuroimaging

Antibodies

Antibodies used	Details for antibodies used are available in Supplementary Table 2 as part of the manuscript files.
Validation	Antibodies were validated by manufacturers and available for each clone on their website, links for each antibody is provided in Supplementary Table 2.

Human research participants

Policy information about studies involving human research participants

Population characteristics	No information regarding the demographic of the human blood donors are known to the investigators.
Recruitment	The donors were recruited independently by the Singapore Health Sciences Authority as platelet blood donors who are otherwise healthy. Investigators receive residual blood cones that are otherwise discarded and are blinded to any profile of the blood donors. Singapore Health Sciences Authority obtains informed consent for the use of residual cone blood in

	research. There is no compensation for participants. There may be self selection bias among donors but this would otherwise not impact the results.
Ethics oversight	Institutional Review Board at National University of Singapore (NUS) and at Agency for Science, Technology and Research (A*STAR), Singapore.

Note that full information on the approval of the study protocol must also be provided in the manuscript.

Flow Cytometry

Plots

Confirm that:

☒ The axis labels state the marker and fluorochrome used (e.g. CD4-FITC).

☒ The axis scales are clearly visible. Include numbers along axes only for bottom left plot of group (a 'group' is an analysis of identical markers).

☒ All plots are contour plots with outliers or pseudocolor plots.

☒ A numerical value for number of cells or percentage (with statistics) is provided.

Methodology

Sample preparation	Samples were obtained by plasmapheresis and PBMCs were isolated from residual cone blood by Ficoll density centrifugation. Details are available in the methods section.
Instrument	The data was acquired on LSR Fortessa or X-20 from Becton Dickinson. (BD).
Software	The data was collected using FACS DiVa software (BD) and analyzed on FlowJo Software (Treestar, BD).
Cell population abundance	We did not sort cells.
Gating strategy	Please see Extended Data Figure 2. In brief, FSC and SSC gates were set for lymphocytes, followed by H vs W gates for singlets in both FSC and SSC. Next we gated for Live cells, followed by CD3+ T cells and distinct gates for CD4 and CD8 T cells. Subsequently CTV, IFNg, IL-17 and TNFa gates were set within the CD4 and CD8 T cell populations, using unstimulated cells (negative) and PMA/ionomycin stimulated cells (positive) to define the boundaries between negative and positive cells.

☒ Tick this box to confirm that a figure exemplifying the gating strategy is provided in the Supplementary Information.
